# Recent Advances in Corrosion Inhibition of Bonded NdFeB Magnets

**DOI:** 10.3390/ma17112475

**Published:** 2024-05-21

**Authors:** Gregor Primc, Miran Mozetič

**Affiliations:** Department of Surface Engineering, Jozef Stefan Institute, Jamova cesta 30, 1000 Ljubljana, Slovenia; gregor.primc@ijs.si

**Keywords:** permanent magnet, NdFeB, corrosion inhibition, liquid polymer, phosphorylation, metallic coating, composite coatings

## Abstract

Bonded permanent NdFeB magnets are useful in numerous applications, including electric vehicles, and the demand is steadily increasing. A major drawback is corrosion due to inadequate wetting of the magnetic particles by liquid polymers such as polyphenylene sulfide or polyamide. Recently reported methods for corrosion inhibition are summarized, and their applicability is critically evaluated. The phosphorylation of magnetic particles inhibits corrosion but does not enable appropriate properties in harsh environments. The same applies to metallic coatings, which usually contain aluminum and zinc. Advanced epoxy adhesives are a promising solution, although some authors have reported inadequate corrosion resistance. The application of composite coatings seems like an appropriate solution, but the exact mechanisms are yet to be studied.

## 1. Introduction

Permanent magnet drives are crucial in many technologies and products, including modern electric and hybrid vehicles. As the strongest widely available permanent magnet material, Nd_2_Fe_14_B-based magnets (often referred to as NdFeB) have been the focus of intense research in basic and applied science as well as manufacturing. NdFeB magnets can be manufactured in several forms, including sintered and bonded. The sintered material has limited applications because of its fragility and poor corrosion properties. Polymer-bonded magnets have gained rising importance due to their applications in electric motors, where shape design flexibility and high-energy products are simultaneously required [1]. NdFeB isotropic bonded magnets are fabricated by injection molding and compression bonding, with a limited magnet powder loading fraction of up to about 65 and 80 vol.%, respectively. Additive manufacturing is an alternative to standard technologies for synthesizing polymer-bonded magnets [2]. A comprehensive article on the applicability of NdFeB composite magnets produced by additive manufacturing was published by Li et al. [3].

Whatever the manufacturing procedure, the technological challenge is using as little polymer as possible and, even so, ensuring reasonable mechanical and corrosion properties of bonded magnets. The inadequate mechanical properties of bonded magnets have been explained by the insufficient wetting of magnetic powder upon interacting with liquid polymers. One of the first reports about inadequate adhesion between the magnet powder and the polymer matrix was by Hemrick et al. [4]. The authors cut the composite material and examined the tensile-fractured surfaces. Their results were persuading—the fracture site was on the boundary between the magnetic particles and polymer. Indeed, the polymer does not wet the magnetic particles when using standard techniques for synthesizing composites. Despite the application of different binders, the problem of poor wetting has remained unresolved until nowadays.

The inadequate mechanical properties of bonded polymers are not the only limiting factor for the application of the magnets in harsh environments. The other is corrosion. Namely, the NdFeB material tends to corrode by forming various oxides and hydroxides. In fact, Nd is among the materials with the highest standard enthalpy of formation. At the value of −1808 kJ/mole, the standard enthalpy of formation for neodymium oxide is even larger than for alumina. The enthalpy is large enough that the fine-powdered NdFeB materials glow red-hot during oxidation in the air [5].

Both the inadequate mechanical properties and the poor corrosion resistance of bonded NdFeB magnets are due to the chemical composition and structure of NdFeB materials, inadequate wetting of the molten polymer, and little chemical interaction at the interface between magnetic particles and the polymer matrix. Therefore, either the magnetic particles, the synthesized magnets, or both should be coated with a protective coating. Numerous authors tried different techniques for the deposition of such coatings, and this paper reviews the recent advances; only papers and patents published after 2020 were considered.

Several methods for detecting corrosion resistance have been invented and are nowadays frequently used in both scientific studies and industrial praxis. Among them, the neutral salt spray (NSS) test is beneficial for qualitatively determining the performance of anti-corrosion coatings. Details about this method are provided in the ISO 9227 specification [6]. Briefly, a sample is placed into a chamber where a fog containing dense and small droplets is created by the atomization of a water solution of salt (NaCl). The atomization should be performed by spraying through a nozzle using pressurized air in the range of pressures between 0.7 and 1.7 bar above the ambient pressure so that the droplets are constantly injected into the chamber at a large size. The salt concentration in previously deionized water should be around 5% (4.5–5.5 g/L), the pH should be adjusted close to neutrality (6.5–7.2), and the electrical conductivity should be below 20 μS/cm according to the ISO standard. The droplets are small enough to levitate in the atmosphere and adhere to the surface of the tested specimen, thus providing a continuous supply of salted water. NaCl dissociates in deionized water, so the neutral salt spray test provides a continuous supply of Cl^−^ ions, which are renowned for their corrosivity. The corrosion resistance of a coating is evaluated as the time the specimen spends in the NSS chamber before the first visible corrosion appears. The NSS method is qualitative, simple, and thus reliable. Other methods for evaluating corrosion inhibition include changes in the specimen weight and functional properties, as well as advanced methods for material characterization.

## 2. Scientific Literature

The scientific literature on the synthesis, characterization, and modification of the surface properties of magnetic NdFeB materials is vast. Perhaps the first scientific paper was published by the inventors of high-density sintered magnets [7]. Simultaneously, another team developed a method for synthesizing melt-spun nanocrystalline NdFeB magnets [8]. Later, many review articles on different scientific aspects of bonded NdFeB magnets were published, including [1]. The recent scientific literature on methods for suppressing corrosion of NdFeB material is reviewed in this section.

### 2.1. Modification of the Magnetic Powder

**Figure 1:**
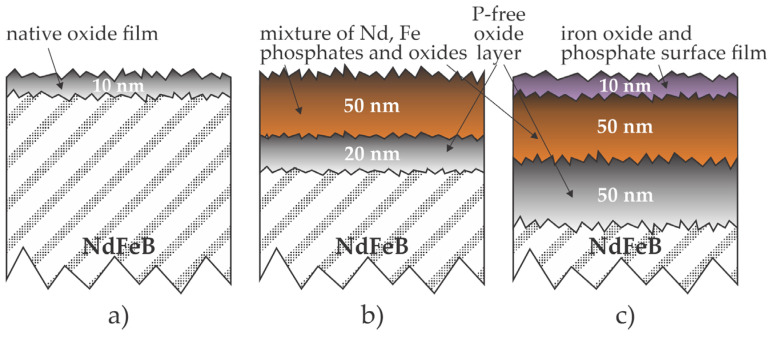
Interaction of phosphoric acid with NdFeB materials, as deduced from the results reported by Shimba et al. [9]. (**a**) Untreated NdFeB is covered with a thin oxide film rich in Nd; (**b**) about 50 nm thick surface film on NdFeB treated in the acid at 120 °C is rich in phosphates and oxides, and the intermediate oxide film is free from phosphorus; (**c**) samples treated in acid solution at 300 °C assumed the surface film free from Nd, followed by an Nd-rich film of phosphates and oxides, and a relatively thick intermediate film rich in oxides is formed between the P-rich film and the bulk NdFeB.

An ideal solution to inhibit the corrosion of bonded NdFeB magnets is the deposition of a corrosion inhibitor onto the powdered magnetic material prior to mixing with a polymer. Shimba et al. [9] reported the application of phosphoric acid to form a corrosion barrier. The dysprosium-free anisotropic NdFeB magnetic powder was prepared in the same laboratory by dynamic hydrogenation disproportionation desorption and recombination method, which is among the standard techniques for synthesizing NdFeB ingots. The ingots were sieved to obtain powder material. The orthophosphoric acid was diluted in propanol, so the acid-to-magnetic material ratio was below 0.1%. Such a low ratio did not cause a measurable modification in the powder structure as deduced by X-ray diffraction (XRD). The suspension was heated to different temperatures to stimulate chemical reactions and diffusion in the surface films. The Fe chemical state in the surface film of several nm thicknesses was determined from the high-resolution X-ray photoelectron spectroscopy (XPS) spectra. The samples treated with the acid at room temperature contained almost identical concentrations of the FeO and Fe_2_O_3_ phases, and the concentration of Fe bonded to phosphorous was below the detection limit (a few at.%). The samples treated with the acid at 120 °C contained about 1/3 of FeO and 2/3 of FePO_4_, and those treated with the acid at 300 °C revealed 2/3 of Fe_2_O_3_ and 1/3 of FePO_4_. No explanation for such an evolution of the iron chemical bonds in the surface film was provided, but the deconvolution of the high-resolution Fe XPS spectra is not trivial. Depth profiling was performed by the Auger electron spectroscopy (AES) method, which revealed a thickness of the oxygen-rich film of less than 10 nm for the untreated samples; on the other hand, for samples treated with acid at 120 and 300 °C, the thickness was 70 and 90 nm, respectively. Interestingly enough, the thickness of the P-rich film was about 50 nm for both samples treated with phosphoric acid, irrespective of the bath temperature. AES depth profiling also showed some segregation of Nd in the surface film, especially at 120 °C, where the Nd/Fe ratio was over 1 in the range of depths between 5 and 30 nm. The corrosion resistance was determined by measuring the coercivity change after immersing the powder in 50% long-life coolant for 100 h at 150 °C. The as-synthesized samples lost over 40% of the coercivity, but the corrosion resistance increased monotonously with increasing temperature of the acid solution and reached a change in coercivity of 28, 10, 4, 2, and 1% at the temperatures of 120, 200, 250, 300, and 350 °C, respectively. The method disclosed by Shimba et al. [9] is illustrated in Figure 1.

Chen et al. [5] also used phosphoric acid but mixed it with isopropyl tris-(dioctyl pyrophosphate acyloxy) titanate (ITDT). Coarse NdFeB powder was mixed with zirconia balls in a ball-milling tank, and the chemicals were added. The ball-milling process was performed at room temperature in an argon atmosphere, and the fine powder was cleaned by rinsing it with pure ethanol and vacuum drying at a temperature of 50 °C for 3 h. The powder was milled in phosphoric acid (ITDT), and the mixture of both chemicals was heated in the air at 250 °C. The fine powder milled in ethanol burned abruptly when the temperature reached 250 °C; the same applies to the fine powder milled in ITDT, except that the burning was delayed. The fine powder, which was milled in phosphoric acid only, and the acid with ITDT did not burn. The corrosion resistance of all samples was determined by measuring the weight gain using thermal gravimetry. The weight gain after obtaining the temperature of 230 °C (before burning) was 2, 1, 0.3, and 0.1% for samples milled in ethanol, ITDT, phosphoric acid, and a mixture of ITDT and phosphoric acid, respectively. The authors explained the excellent corrosion resistance of the latter sample by adhering the PO_4_ radicals to the Fe_3_^+^ and/or Nd_3_^+^ surface ions and covalently bonding the ITDT onto the PO_3_OH radical. The explanation is different from that provided by Shimba et al. [9], probably because Shimba et al. treated the NdFeB in phosphoric acid at elevated temperatures, and appropriate results (change in coercively below 2%) were observed only after treating the powder at temperatures above 300 °C. Unfortunately, Chen et al. [5] did not explicitly report the bath temperature, but it is reasonable to assume they performed the treatment with the acid at room temperature. The protective film’s thickness and composition depend significantly on the bath temperature, as shown by Shimba et al. [9].

### 2.2. Adhesive Coatings

**Figure 2:**
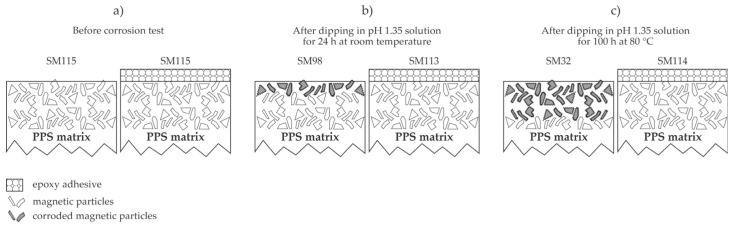
The beneficial effect of 3M Scotch-Weld DP100 adhesive on printed NdFeB magnets of 5-mm thickness. (**a**) As-synthesized both uncovered (**left**) and covered (**right**) NdFeB magnets are free from corroded magnetic flakes and exhibited the saturation magnetization (SM) of 111.5 emu/g; (**b**) after dipping in the acid solution for 24 h at room temperature, the uncovered NdFeB magnet partially corroded, so the saturated magnetization dropped to 98 emu, while the covered magnet remained intact; (**c**) after dipping in the acid solution for 100 h at 80 °C, the uncovered NdFeB magnet corroded significantly, so the saturated magnetization dropped to 32 emu, while the covered magnet remained intact.

A trivial solution for protecting bonded NdFeB magnets is to cover them with adhesive coatings of about 10 µm thickness. The adhesive should not be permeable for a corrosion medium. Paranthaman et al. [10] synthesized bonded magnets by extrusion-based additive manufacturing. They used a large-area additive manufacturing printer for the rather fast deposition of NdFeB–polyphenylene sulfide (NdFeB–PPS) composites. The extrusion rate of the printer was as large as 25 mm/s, and the substrate moved at a fast speed, so a layer thickness of 5 mm was achieved. The as-synthesized magnets were coated with two different adhesive layers, either 3M Scotch-Weld DP100 or J-B Weld epoxy coating. The stability of the as-synthesized bonded magnets and those covered with adhesive films was studied after annealing at different temperatures. The magnetic flux loss, which is supposed to be mainly due to oxidation of NdFeB, for uncovered magnets after annealing in the air at 100 °C was 1.0, 0.9, and 0.7% for uncoated magnets and magnets coated with 3M Scotch-Weld DP100 or J-B Weld epoxy coating, respectively, so the coatings were found beneficial. However, the flux loss after annealing at 127 °C showed the opposite trend, i.e., 2.6, 3.5, and 5.8% for uncoated magnets and magnets coated with 3M Scotch-Weld DP100 or J-B Weld epoxy coating, respectively. Various annealing temperatures up to 200 °C were probed, and the flux loss was not monotonously dependent on either the annealing temperature or the type of adhesive used as coverage. The benefits of covering magnets with 3M Scotch-Weld DP100 adhesive were well-expressed after performing the corrosion tests by dipping the not-annealed samples into an acid solution at pH 1.35. The saturation magnetization was measured in order to evaluate the corrosion inhibition. The as-synthesized samples exhibited a saturation magnetization of 111.5 emu/g. After dipping at room temperature for 24 h, the saturation magnetization dropped to 98 emu/g for uncoated samples and even slightly increased to 113.3 emu/g for the samples coated with 3M Scotch-Weld DP100 adhesive. It was as low as 32 emu/g for uncoated samples after dipping into the acid solution at 80 °C for 120 h, while samples coated with 3M Scotch-Weld DP100 adhesive remained intact since the saturation magnetization after 100 h remained as large as 113.7 emu/g. The method for suppressing the corrosion, as reported by Paranthaman et al. [10], is illustrated in Figure 2. Indeed, the scotch adhesive of a thickness of about 10 µm prevents the acid solution from coming into contact with the magnetic material, so it is a valuable solution for preventing corrosion, at least up to the temperature of 80 °C. This is due to this type of adhesive’s composition and low viscosity, which allow for easy dispensing and filling of any gaps or pores on the substrate surface. Namely, the non-cured epoxy adsorbs adequately even on untreated PPS surfaces. Treatment of PPS polymers by reactive species from non-equilibrium gaseous plasma causes a further increase in adhesion strength [11]. The unfavorable results reported by Paranthaman et al. [10] at larger temperatures between 100 and 200 °C may be explained by structural modification of this adhesive, as the highest recommended curing temperature is 93 °C. The same adhesive was also reported to be beneficial for coating hybrid magnets containing both NdFeB and SmFeN because the magnetic flux decreased by much less than 1% of the value measured for uncoated magnets [12].

**Figure 3:**
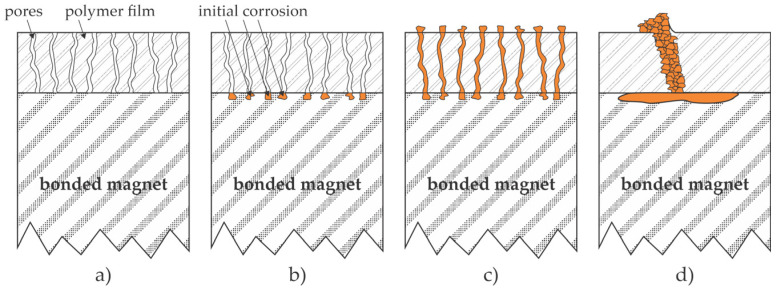
Mechanisms leading to the appearance of corrosion products on the surface of epoxy resin deposited on the NdFeB magnets, according to Yang et al. [13,14]. (**a**) The water with Cl^−^ ions slowly penetrates the pores in the epoxy film; (**b**) the initial degradation stage is corrosion at the interface between the magnet and polymer; (**c**) larger pores enable diffusion of corroded magnet material onto the polymer surface; (**d**) small pores do not enable diffusion of corroded material, but the corroded magnet causes swelling of the polymer film and eventually peeling off.

An epoxy coating to improve the corrosion resistance of bonded NdFeB magnets was also applied by Yang et al. [13]. The exact composition of the epoxy resin was not reported. Scanning electron microscopy (SEM) images revealed rough surfaces of as-synthesized bonded magnets with dense gaps of width up to a few µm. Yang et al. [13] mixed an epoxy resin lacquer with an electrostatic dilutant, sprayed it onto the as-synthesized magnets, and thus covered the gaps on the magnet surface. However, the cured resin with a thickness of about 10 µm contained numerous pores and holes. Furthermore, the adhesion between the bonded magnet and epoxy layer obtained by spraying was inadequate, as revealed from the cross-cut adhesion images. The coated samples were tested for corrosion properties using a neutral salt spray (NSS) test. The as-synthesized magnets corroded extensively even after 24 h, but the visible corrosion of the samples sprayed with epoxy resin appeared only after 72 h of the NSS test. Still, the corrosion properties of the sprayed magnets were inadequate, which the authors explained by the penetration of the corrosive medium through pores, chemical interaction with the magnetic material, and migration of corrosion products on the surface of samples sprayed with epoxy resin. The effect is illustrated in Figure 3a–c.

### 2.3. Deposition of Adhesive by Electrophoresis

Yang et al. [13] also proposed an alternative method for the deposition of epoxy resin—they employed electrophoresis. The authors used a water solution of a cathode electrophoresis resin, a solvent, and a pigment slurry. The pH was adjusted to 6.3, and the conductivity of the solution was about 0.12 S/m at 30 °C. The voltage of 110 V was applied for 2 min for the deposition of a resin film with a thickness of about 13 µm. The adhesion between the resin deposited by electrophoresis and the magnets was adequate, definitely much better than the adhesion of the same resin deposited by spraying. Furthermore, the corrosion properties improved compared to sprayed samples since visible corrosion upon NSS tests appeared after 144 h. Still, the long-term protection was found inadequate. The authors attributed the differences in the corrosion behavior between sprayed and electrophoretic depositions to smaller pores and/or more densely packed resin clusters in the polymer film, as illustrated in Figure 3.

In another paper [14], the same team proposed a double-layer coating obtained by deposition of the epoxy film by electrophoresis, followed by deposition of the same resin by spraying. In this paper [14], the authors also reported the pore size in polymer films, which was determined by measuring the adsorption curves of nitrogen. The pore size peaked at about 14 nm for films obtained by both deposition methods. The authors also mentioned the super-hydrophobic character of the polymer film using this double-layer deposition technique, which should, according to the authors, prevent chlorine ions and water molecules from penetrating into the substrate. Even so, the corrosion properties were barely adequate, as visible corrosion appeared after 216 h of the NSS test. The authors [14] proposed a corrosion model different from that in [13]. Specifically, they attributed the appearance of visible corrosion products on the surface of the adhesive coating to slow penetration of the corrosive species through the pores, thus triggering corrosion of the magnetic material at the interface between the NdFeB and the polymer resin. The corrosion products accumulate at the interface, which leads to bulging and peeling of the coating; this should explain the visible corrosion observed after 216 h of the NSS test. Both mechanisms (proposed in [13] and [14]), which result in the appearance of the corroded magnet material on the surface of the polymer coatings, are illustrated in Figure 3. The illustrations are inappropriate for some types of epoxy coatings, such as 3M Scotch-Weld DP100 adhesive, which is supposed to be free from pores [10].

### 2.4. Deposition of a Composite Coating

**Figure 4:**
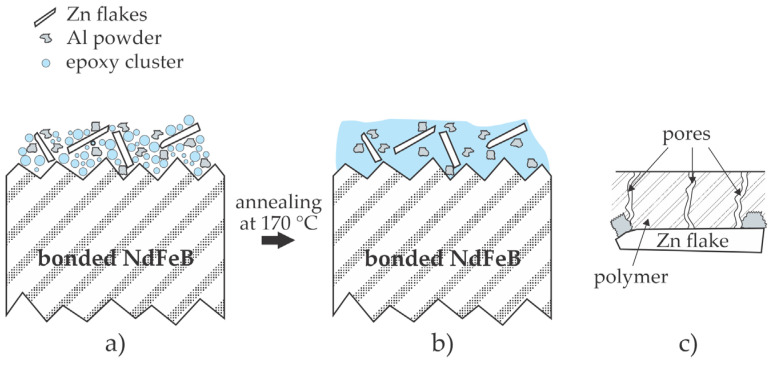
Illustration of the beneficial effect of Zn and Al powder admixes in the epoxy coating on the corrosion resistance of bonded NdFeB magnets. (**a**) The as-deposited composite film contains pores and gaps; (**b**) the annealing at 170 °C enables molten epoxy to fill the gaps; (**c**) any pore will likely terminate at the metallic particle.

A step further from epoxy coatings is the deposition of composite coatings. Yang et al. [15] mixed epoxy resin with Zn and Al powders in order to prepare coatings that exhibit adequate corrosion resistance for bonded NdFeB magnets. They added Zn and Al powders to the electrophoresis bath. The deposition occurred in a water mixture with epoxy paint, a cation electro-coat resin, a solvent, a cation electro-coat paste, and the Zn and Al powders. The as-deposited layer was cured in a furnace at 170 °C for 20 min. The authors showed that this procedure enabled the deposition of a coating with a relatively uniform thickness of about 30 µm, and the polymer component in the coating efficiently filled the gaps between the Zn and Al particles after the curing. The samples were subjected to the NSS test, which exhibited no corrosion even after almost 1000 h of exposure. The composite coating prevented contact between the corrosive medium and the magnetic substrate and did not influence the magnetic properties of the bonded NdFeB magnets. No adhesion test was performed; however, it seems that the epoxy polymer wetted the magnet material adequately and represented an adhesive between the NdFeB substrate and the Zn and Al particles. It is reasonable to assume that the pores appeared in the epoxy layer during deposition by electrophoresis, the same as in [13], but the pores might terminate at the surface of the Zn or Al particles. The effect is illustrated in Figure 4.

### 2.5. Deposition of Metallic Coatings

**Figure 5:**
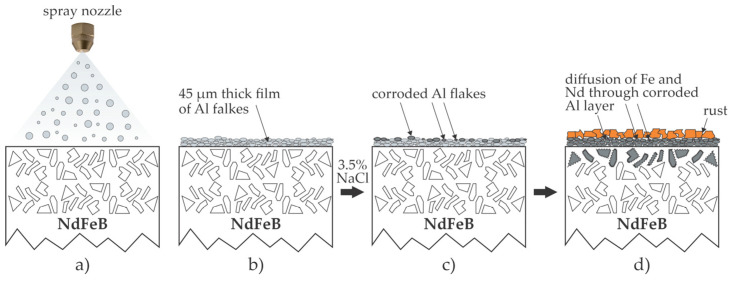
The effect of Al coating on the corrosion resistance of NdFeB magnets. (**a**) The magnets are sprayed with Al particles of predominantly spherical shape and various diameters; (**b**) a film containing densely packed Al flakes covers the magnet; (**c**) corrosion of Al flakes occurs upon interaction with Cl^−^ ions; (**d**) after prolonged exposure to the corrosion medium, the diffusion from the magnet towards the surface is triggered, resulting in the appearance of rust on the surface of the corroded Al film.

Gao et al. [16] deposited several 10 µm thick aluminum films by spraying aluminum spheres with a diameter of roughly 10 µm onto NdFeB magnets. The substrate temperature was 150 °C during spraying, so well below the Al melting point. Aluminum spheres somehow flattened when impinging the substrate surface, forming a rather uniform film free from macroscopic voids. The adhesion of the Al film was adequate, and so were the magnetic properties of coated NdFeB magnets. The coated samples were tested for corrosion properties using an NSS test. The aluminum coating was beneficial since visible rust appeared on the Al-coated samples after about 100 h in the testing chamber. Uncoated samples were heavily corroded, and the corroded materials started to peel off. The corrosion of metals upon exposure to a 3.5 wt.% NaCl water solution was attributed to the diffusion of Cl^−^ ions through the native oxide films. The aluminum coating was compact enough to prevent the diffusion of chlorine into the NdFeB for the first tens of hours, but the authors explained the corrosion after prolonged treatment in the test chamber by the conversion of Al oxides into chlorides, resulting in the failure of the Al film after prolonged testing times. The corrosion rate was estimated by measuring the weight loss, and it was almost an order of magnitude lower for Al-coated than for uncoated NdFeB magnets after 200 h. The method reported by Gao et al. [16] is illustrated in Figure 5. The water solution of NaCl is polar enough, so it does not penetrate through the film of Al flakes. The penetration occurs only after significant corrosion of the Al flakes. As the solution diffuses inside the film of corroded Al flakes and comes into contact with the magnetic material, the diffusion of Fe and Nd ions through the layer of the corroded Al flakes occurs. The final effect is the formation of the corroded magnetic material on the top of the film of corroded Al flakes. Although the authors did not report it, the cracks in the metal film are likely to occur during corrosion since the volume of the corroded Al flakes is much larger than the volume of the as-deposited Al flakes. The corrosion is thus significantly postponed by the deposition of an Al film using the spray method.

The Al flakes may be replaced with Zn and Al powder, as reported by Yang et al. [17]. The authors sprayed Zn and Al powder together with silane. The silane was pre-mixed with ethanol and water, and the metallic powders were added to the mixture upon continuous stirring, which lasted several hours. Surfactants were also added to the mixture to prevent the powder’s aggregation or agglomeration, but the authors did not mention the details. The well-stirred liquid was then sprayed onto the bonded NdFeB magnets. The as-sprayed samples were annealed in an oven at 120 °C for 15 min. The final procedure was solidification by infrared heating at 230 °C for 45 min. SEM micrographs revealed densely packed flakes of Zn and Al of irregular shape and size, as well as smaller clusters of Si. According to the authors, some adhesive was also present in the coating, but it was difficult to identify it from the SEM micrographs. The aim of the Zn and Al particles was to inhibit corrosion of the magnetic material because of the screening effect—preferential corrosion of the Zn and Al was expected. Corrosion products became visible after 264 h of the NSS test, and the authors compared the results with some other coatings of similar thickness, for which the corrosion was visible after 48, 96, and 120 h for bonded NdFeB magnets coated with zinc, aluminum, and Zn/Co, respectively. The coating did not change the magnetic properties much because the magnetic flux decreased by much less than 1% of the value measured for uncoated magnets.

## 3. Patent Literature

The technological importance of corrosion inhibition is exceptionally high, so the patent literature on the methods for corrosion inhibition of NdFeB is vast. Only selected recent patents or patent applications are reviewed. The Japanese inventors (some of whom coauthored the scientific paper [7]) filed the patent application in 1983, which was published as [18] in 1984. The American team that coauthored [7] filed the patent application in 1982, which was published in 1983 [19]. Another early patent application by the same team was filed in 1983 [20] and granted in different countries in the following years. The first patent applications specifically mentioning NdFeB were filled out in the late 1980s of the last century and published a year or two after submission to the patent office [21,22,23]. Recent patents are reviewed below.

### 3.1. Deposition of a Metallic Film

**Figure 6:**
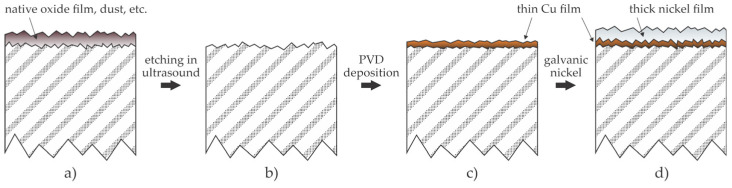
Illustration of a method for the deposition of metallic films by sputtering and electrochemical deposition. (**a**) As-synthesized magnets are covered with a film of impurities; (**b**) the impurities are removed by acid treatment in an ultrasound bath; (**c**) a thin copper film is deposited by sputtering in a high vacuum chamber; (**d**) a thicker nickel film is deposited by electrochemical methods.

Corrosion inhibition by the deposition of metallic films was also a subject of several recent patents. For example, ref. [24] discloses the method for corrosion suppression by deposition of thin copper and nickel films. The NdFeB magnets are first cleaned by ultrasonic treatment in a pickling solution. The cleaned magnet surface is then covered with a thin copper film, which is preferably deposited by magnetron sputtering. The next step is the electrochemical deposition of a nickel film. The inventors claim that the thin metallic films on the surface of the NdFeB magnets cause optimal corrosion resistance and do not result in a loss of magnetic properties because the magnetic shielding generated by the plating layers is marginal. The examiner’s opinion on this patent application has yet to be published. The methods disclosed in [24] are illustrated in Figure 6. As-synthesized magnets are covered with a layer of impurities, which consists of the native oxide film formed on the NdFeB flakes, as already shown in Figure 1a, as well as dust, organic impurities, etc. The removal of surface impurities is necessary for reasonably good adhesion of a metallic film deposited by magnetron sputtering. The copper atoms condensing on the substrate surface will form a well-adhered, thin metallic layer. The deposition rate by sputtering is limited, so this technique is not practical for depositing films of thickness greater than a few µm, so the final step is galvanic (electrochemical) deposition of nickel. Direct galvanic deposition (without an intermediate copper film) is not feasible because it is challenging to ensure good adhesion between the substrate and the nickel film. The electrical conductivity of NdFeB is high, but bonded magnets only exhibit fair conductivity [25]. Furthermore, nickel films deposited by galvanic deposition are loosely bonded to the surface of the polymer [26], which binds the magnetic powder in magnets. The drawbacks of the deposition of metallic films by surface condensation of atoms from the gas phase upon physical vapor deposition (PVD) will be discussed in Section 4.

### 3.2. Deposition of a Composite Film

Patent CN202310303359 [27] discloses a method for coating the magnets with a composite layer that contains epoxy adhesive and hexagonal boron nitride (BN) particles. The BN particles are first processed to improve their dispersibility in the electrophoresis solution. The solution contains epoxy adhesive, polydopamine, and modified BN particles and is stirred well before the magnets are immersed in the bath to obtain a finely dispersed suspension. The role of polydopamine is to stick to the BN particles and thus represent an intermediate film between the epoxy matrix and the inorganic particles. The thin film deposited on the magnets is thus a composite containing finely dispersed BN particles in a polymer matrix. The corrosion resistance of the epoxy composite coating formed by adding polydopamine-coated BN particles into the electrophoresis solution through cathode electrophoresis is greatly improved, and the filler preparation process is simple and environment-friendly. The examiner’s opinion is that [27] is not patentable in view of [28,29]. Nevertheless, the beneficial effect of the BN particles dispersed in the epoxy adhesive is illustrated in Figure 4c. The particles dispersed in the polymer film terminate the pores and other structures in the polymer film. The corrosive medium will penetrate the pores and trigger corrosion sooner or later if the standard epoxy coating is deposited on the magnet surface, as illustrated in Figure 3. However, finely dispersed particles in the epoxy adhesive will suppress the propagation of the corrosive medium through the coating, especially if they are chemically inert, like boron nitride. A technological challenge, however, is the deposition of the composite film free from aggregates, as will be discussed in Section 4.

### 3.3. Surface Oxidation or Nitriding

US2023282414 [30] discloses a method for the formation of a protective oxide or nitride film by heating the NdFeB magnets in an oxygen or nitrogen atmosphere at elevated temperatures. In one embodiment, the magnets are placed in a high-vacuum chamber, which is pumped down to a pressure of 2 × 10^−5^ mbar. Oxygen is then introduced into the chamber at a rate of 800 sccm during continuous pumping, and the samples are kept at 350 °C. The reaction time was as long as 3 h, probably because of the low-pressure conditions and/or limited diffusion of ions through the oxide or nitride film. A compact oxide film of a thickness of about 1 µm was formed on the NdFeB magnets. The NSS tests were performed after the oxidation, and the corrosion current was marginal compared to the current measured for non-oxidized magnets. The nitridation was performed using ammonia as the nitrogen source. Using ammonia instead of nitrogen is beneficial because it suppresses oxidation, so an almost pure and compact nitride film is obtained on the metallic surface [31]. The vacuum chamber was first evacuated down to 3 × 10^−7^ mbar to ensure a negligible amount of gases comprised the residual atmosphere. After thorough evacuation, the ammonia was introduced during continuous pumping with a flow rate of 80 sccm and a sample temperature of 400 °C. A compact nitride film of 0.3 µm thickness was formed during treatment with ammonia for half an hour. The examiner found the patent application inappropriate in view of WO2009/041639 [32].

The formation of oxide or nitride films on metallic surfaces by exposure to oxygen or nitrogen-containing gases requires elevated temperatures because the diffusion of ions through compact oxide or nitride films is limited at room temperature, and only a native film would appear, as illustrated in Figure 1a. An alternative is to partially avoid the diffusion and implant appropriate dopants (oxygen or nitrogen ions). US2022154327 [33] discloses an ion implantation method for treating NdFeB magnets. Either oxygen or nitrogen ions were implanted. In the case of oxygen implantation, a thin and compact oxide film was created, while in the case of nitrogen implantation, nitrogen formed iron nitride, which effectively protects the magnet material from oxidation. The inventors reported no N- or O-rich coating delamination from the NdFeB materials. The preferred kinetic energy of ions impinging the magnet surface is about 25 keV, and the preferred dose is about 2.5 × 10^21^ m^−2^. The preferred pre-treatment of the magnets was sandblasting with alumina powder and rinsing with ethanol. The treated magnets were subjected to an accelerated aging test in the air at 60 °C and 90% relative humidity for 7 days, and no visual corrosion was observed. The untreated magnets were quite corroded after the same test. The Japanese and Korean patent offices have already granted this patent as JP7232302(B2) and KR102536438(B1), respectively. Ion implantation involves numerous effects and is still a subject of scientific interest [34], although it has been used for doping solid materials for decades [35]. The projectiles cause radiation damage (displacement of atoms in the surface film reached by the ions) as well as sputtering (removal of surface atoms from the solid material) by kinetic effects. At room temperature of the sample, the sputtering prevails at kinetic energy up to a few keV and the implantation at larger energies like 25 keV, which is preferred in US2022154327 [33]. The penetration depth for 25 keV nitrogen ions is several 100 nm, and a gradual decrease in the N concentration in the metallic film is observed [36]. The annealing of implanted materials may change the distribution of implanted ions.

**Figure 7:**
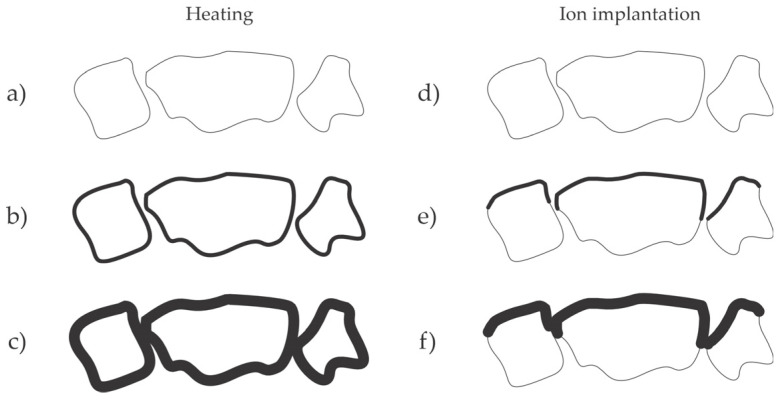
Formation of the oxide or nitride film upon heating (**a**–**c**) and ion implantation (**d**–**f**). (**a**,**d**) untreated magnetic particles contain only the native surface oxide film; (**b**,**e**) short treatment times cause a thicker layer; and (**c**,**f**) long treatment times cause a thicker surface layer.

Both heating in a controlled atmosphere and ion implantation cause the formation of the oxide or nitride films, but the crucial difference is that low-temperature ion implantation causes film formation only on the surface facing ions on the top-most magnetic grains, whereas thermal treatments cause a more isotropic layer, which often forms on the entire grain surface. The effect is illustrated in Figure 7. In both cases, the oxide or nitride film causes smaller gaps between the grains because it is thicker than the original metal surface film.

Another alternative is heating NdFeB magnets in an oxidizing atmosphere containing organic vapors, as disclosed in US2023178274 [37]. A variety of alcohols and organic acids provide excellent results. The preferred temperatures are between 200 and 300 °C, and the preferred treatment time is 1 h. The thickness of the corrosion-protective film deposited according to the methods of the invention disclosed in [37] is around 1 µm. The samples are heated in a vacuum oven, and the partial pressures of oxygen, water vapor, and organic vapors are preferably a few 10, 1, and 0.1 mbar, respectively. The inventors claimed that the addition of organic vapor caused the enhanced formation of the hematite on the surface of magnetic material. The Chinese patent office has already granted the patent as CN112259359(B). The treatment should result in a similar effect as illustrated in Figure 7a–c, except that the temperature for obtaining the same thickness of the oxide film is lowered because of the beneficial effects of the organic vapors.

### 3.4. More Complex Strucures

A method for the deposition of a super-hydrophobic film is disclosed in CN116190041 [38]. A three-dimensional network structure formed by connecting Si−O−Si bonds is generated on the NdFeB surface. The bonds serve as a glue between the oxides and hydroxides formed simultaneously during the treatment. The oxidation products and the silane bond enhancer form a three-dimensional network structure that is porous. As disclosed in this patent application, the pore dimensions are several µm. The composite film exhibits a super-hydrophobic character, which, according to the inventors, should prevent penetration of corrosive media through the pores. Such penetration is otherwise likely to occur, as illustrated in Figure 3. The inventors reported excellent corrosion resistance of the materials coated according to the invention methods and a minor influence on magnetic performance. Furthermore, they reported a strong binding force between the coating and the substrate.

The utility model CN218786671 [39] discloses a method for depositing a complex coating on an NdFeB substrate. The first step is nickel plating, which uses citrate as the precursor. Next, a copper layer is deposited using the copper pyrophosphate bath. This layer is coated with a thicker copper film using the acid copper plating method. A bright nickel film is deposited on top of the copper, followed by a trivalent black chromium plating layer. The surface of such a sandwich layer is decorated with graphene. The corrosion of the magnets coated using this method was tested by NSS. Visual inspection showed no corrosion of the samples after at least 168 h of the NSS test. The inventors also reported good adhesion of the corrosion-protected film to the magnet substrates.

CN115896768 [40] discloses a method for the deposition of a chromium-free coating on the NdFeB magnets. The as-synthesized magnets are first coated with a layer of zinc and subsequently with a silane coating, which is deposited by galvanic deposition from a bath containing both silanes and titanium salt.

## 4. Discussion and Perspectives

**Figure 8:**
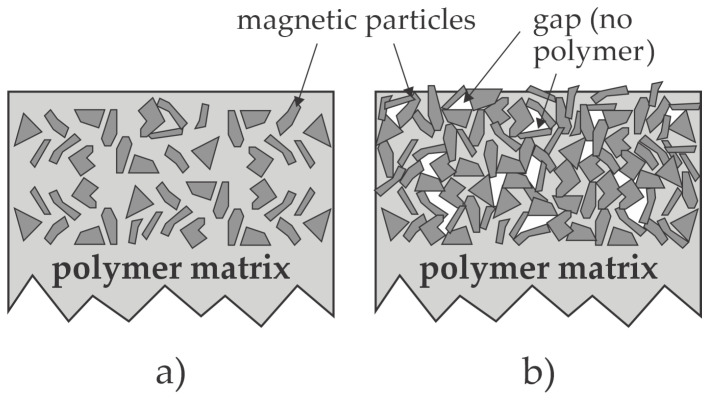
Illustration of the bonded magnets with a high (**a**) and low (**b**) polymer concentration.

Recent progress in the methods for the protection of NdFeB magnets against corrosion indicates encouraging results, but an optimal solution is yet to be invented. The magnetic properties increase with an increasing ratio between the amount of magnetic and polymer materials, so the concentration of magnet particles in the polymer should be as high as possible. On the other hand, the low concentration of polymer may not be sufficient to fill all gaps between the magnetic particles, as illustrated in Figure 8. When using the magnets in a harsh environment, the gaps will be filled with corrosive media sooner or later, and corrosion will occur. The low polymer concentration in bonded magnets also causes direct contact between neighboring magnet particles, as shown in Figure 8b, so the corrosion continues.

A straightforward solution for preventing the corrosion of bonded magnets is to make the magnetic particles insensitive to a corrosive medium. A possible method is illustrated in Figure 1. A layer of phosphates is formed on the surface of magnetic particles, representing a diffusion barrier, so the corrosion is suppressed. Another solution is the formation of oxide or nitride films, as illustrated in Figure 7. The films suppress the corrosion but do not prevent it after using the magnets in a harsh environment.

Another solution for protecting magnet particles from corrosion would be the deposition of an ultra-thin film of a material with optimal anti-diffusion properties. Industry often deposits a film of highly resistant polymer on metallic surfaces. Probably the most sophisticated method is the deposition of the polydimethylsiloxane coating with a thickness of a few 10 nm by plasma polymerization on metallic objects [41]. This technology enables a stable diffusion barrier over months of exposure to the NSS solution. The technology is optimal for protecting macroscopic objects with a smooth surface from corrosion but inappropriate for treating powders because the treatment time would be too long and the uniformity of the film thickness would be questionable.

The magnetic particles in bonded magnets should be wetted perfectly by the molten polymer because non-coated areas on the magnet surface will corrode quickly. The wetting of solid materials is governed by the viscosity of the molten polymer, the surface tension of the liquid polymer, and the surface free energy of the magnetic material. Standard polymers in bonded magnets are polyphenylene sulfide and polyamides. Both exhibit appropriate chemical and mechanical properties, but the adhesion between the magnet material and the polymer matrix is not optimal. From this point of view, polyether ether ketone (PEEK) may be a solution in specific niches (printed magnets, for example) since it provides fairly good interface adhesion [42]. The adhesion between a polymer and a coating depends on the balance between the surface properties of both materials [43], but no recent literature has mentioned any pre-treatment of the magnetic powder for optimization of the surface free energy of NdFeB materials.

An alternative to protecting powders is to deposit a film of corrosion-protective layer on the fabricated bonded magnets. The protective layer should adhere well to the entire surface of the bonded magnets. Various coatings have been proposed, and encouraging results have been reported.

**Figure 9:**
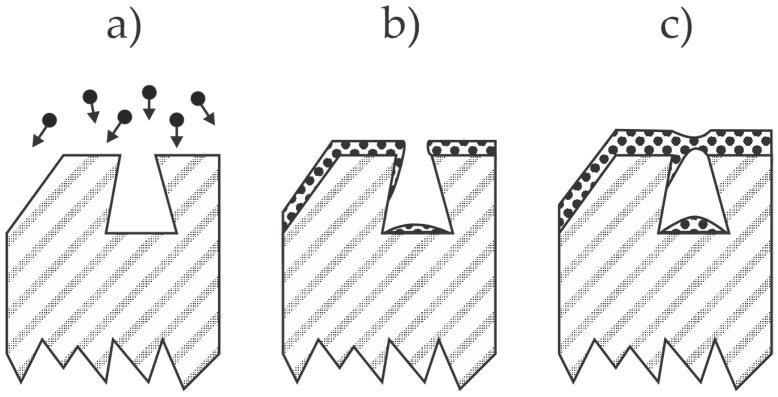
Illustration of the film growth on a rough substrate by PVD or CVD methods for cases where the surface energy is large enough to prevent the formation of particles, so it enables the growth of a uniform film. (**a**) An untreated substrate is subjected to a random flux of condensable atoms or radicals; (**b**) a thin film coats the exposed surfaces after a short deposition time; (**c**) after a long deposition time, the film may cover the gap.

A method for depositing a thin film is the condensation of gaseous atoms or molecules on the substrates. The method is called physical or chemical vapor deposition (PVD and CVD, respectively) and has been widely used in industry. In the PVD process, atoms are released from the source by evaporation or sputtering, and the released atoms diffuse throughout the treatment chamber and finally condense on substrates. Conversely, CVD provides gaseous molecules with high chemical reactivity (often radicals), and they interact chemically on the substrate surface. The exact growth mechanisms reveal these techniques’ complexity on the atomic scale, as was understood decades ago [44]. The defects are also likely to influence growth uniformity [45]. In both PVD and CVD, the adhesion of the formed thin film is adequate as long as the wettability of the substrate is large enough, i.e., the surface free energy of the substrate is large enough to prevent the spontaneous formation of the nanoparticles on the substrate rather than the growth of a uniform film [46]. Furthermore, the surface should be free from impurities, so cleaning is an essential step before using PVD or CVD methods. The technique is useful for coating substrates with smooth surfaces, but as-synthesized bonded magnets rarely satisfy this requirement. The effect of the surface roughness on the coating quality is illustrated in Figure 9 for cases where a film of uniform thickness grows on a smooth surface. The condensable atoms or molecules will not provide a uniform coating on the substrate, but the film thickness will be inadequate in the shaded areas. In fact, pores of dimensions lower than the mean free path of gaseous species will remain free from the deposit and will be covered with a film, and the voids will represent an ideal corrosion site. The deposition should be performed at low pressures because gas-phase agglomeration occurs at elevated pressures, and the deposited films are porous [47].

The effect illustrated in Figure 9 will be less pronounced when using electrochemical methods for thin film deposition, provided the entire surface exhibits similar electrical conductivity and the electrolyte wets the pores on the substrate surface. Wetting is usually ensured by adding appropriate chemicals to the electrolyte solution to decrease the liquid surface tension. However, composite materials like bonded magnets will not provide the same electrical conductivity since NdFeB is conductive and the polymers used in bonded magnets are insulators.

The effect illustrated in Figure 9 does not occur when coating the bonded magnet with a liquid adhesive, provided the surface tension of the liquid is small compared to the surface free energy of the bonded magnets. Different adhesives exhibit various surface tensions, so the adhesive should be chosen carefully. For example, Parantham et al. [10] used two epoxy adhesives and reported adequate results for only one type (see Section 2.2). The adhesives often ensure pore-free coating, but sometimes pores are formed, so corrosion is observed after performing the tests, as illustrated in Figure 3. Furthermore, the polymer coating may age, i.e., lose the appropriate chemical and mechanical properties after extended use. The choice of adhesives is limited to materials that do not absorb large amounts of water.

Mixing liquid polymers with powders to deposit a composite coating may further enhance the adhesive properties. Some authors reviewed in this article reported beneficial results explained by blocking the migration of corrosive medium through the protective film. One feasible explanation is illustrated in Figure 4c. Another may be the chemical interaction between the corrosive medium diffusing through the protective layer. Many polymers are at least slightly permeable for oxidative species, so they slowly diffuse through the polymer film. When they reach the interface with a filler, they will chemically react if the filler is prone to corrosion. The fillers are sacrificed to prevent the oxidative species from reaching the magnet particles.

Composite coatings usually perform well, but the appropriate dispersion of the fillers in the polymer matrix may represent a serious obstacle that limits the choice of polymer/particle combination. Namely, the fillers may not disperse in the liquid polymer, as reported in [27]. Fillers tend to form aggregates in liquids, and the aggregates cannot be avoided even after prolonged stirring. The problems with aggregation may be solved by the addition of a surfactant to the liquid, but the presence of the surfactant may influence the functional properties of the composite protective layer.

Still, the composite coatings for protection of the bonded magnets from corrosion are most promising because the polymer component of the composite coating provides a reasonable diffusion barrier, and the fillers capture the species slowly diffusing through the polymer by irreversible chemical bonding. Many polymers exhibit sufficiently low permeability for corrosion species, so the functional properties of the composite protective coatings will last a long time. The coatings should be thick enough to ensure protection over the magnet’s functional period and thin enough not to suppress the magnetic properties or add to the complexity of the recycling of used NdFeB magnets. Namely, Neodym is a rare earth, and its stock is limited. Furthermore, the extraction and purification of Nd are costly both in economic and ecological terms [48], so the materials are typically recycled rather than disposed of [49].

## 5. Conclusions

Significant efforts have been reported in order to protect the bonded magnets from corrosion, and an optimal solution has yet to be discovered. One possibility is treatment with phosphoric acid to coat the magnetic powder with a layer of oxides and phosphates. Such a treatment suppresses corrosion, but optimal results were reported at a bath temperature above 300 °C, which may not be very practical. The oxidation or nitridation of the magnetic powder by heating it in an appropriate atmosphere will also provide a diffusion barrier and thus suppress the corrosion. The oxide or nitride films could also be synthesized on the surface of magnetic powder by ion implantation, but this solution is not applicable for mass production since it is too slow. The corrosion of the bonded polymers could be suppressed by the deposition of metallic coatings. The coatings could be deposited by spraying, electrochemical methods, or vapor deposition. The metallic coatings efficiently prevent corrosion of the underlying magnetic material, but the coatings themselves are prone to corrosion upon exposure to harsh environments, so such protection does not last long. Another possibility is the deposition of a polymer coating. Unlike metals, polymers will not corrode in ionic liquids such as acids or salts. Polymers differ significantly in terms of viscosity and ability to wet the substrates, and the dried films may be porous. The porosity has a negative impact on the barrier properties for the diffusion of aggressive liquids or even corrosion products. The deposition method will also govern the structure of the dried polymer coating. A promising method is the deposition of composite coatings, i.e., metallic or ceramic particles dispersed in a polymer matrix. A handful of appropriate composite coatings have been reported, but optimization of such coatings still represents a scientific challenge. Once the scientists find the optimal composites, the preparation of such materials on an industrial scale will become a technological challenge. The industry’s main challenges in the niche of composites are developing procedures for synthesizing suitable suspensions free from aggregates, the adhesion of the composite coatings to the surface of bonded magnets, and the durability of the protective coatings. Compatibility with ecological standards and recyclability should be considered, too.

## Data Availability

This is a review paper; no new data were generated.
